# Fractionated administration of irinotecan and cisplatin for treatment of non-small-cell lung cancer: a phase II study of Okayama Lung Cancer Study Group

**DOI:** 10.1054/bjoc.2001.1861

**Published:** 2001-07

**Authors:** H Ueoka, M Tanimoto, K Kiura, M Tabata, N Takigawa, Y Segawa, I Takata, K Eguchi, N Okimoto, S Harita, H Kamei, T Shibayama, Y Watanabe, S Hiraki, M Harada

**Affiliations:** Department of Internal Medicine II, Okayama University Medical School, 2-5-1 Shikatacho, Okayama, 700-8558, Japan; Department of Respiratory Medicine, National Shikoku Cancer Center; Department of Internal Medicine, Kawasaki Medical School, Kawasaki Hospital; Department of Internal Medicine, Chugoku Central Hospital; Department of Internal Medicine, Sumitomobesshi Hospital; Department of Internal Medicine, Okayama Health Foundation Hospital; Department of Internal Medicine, Okayama Red Cross General Hospital

**Keywords:** phase II study, irinotecan, cisplatin, fractionated administration, non-small-cell lung cancer

## Abstract

A phase II study of fractionated administration of irinotecan (CPT-11) and cisplatin (CDDP) in patients with non-small-cell lung cancer (NSCLC) was conducted. Between January 1996 and January 1998, 44 previously untreated patients with stage IIIB or IV NSCLC were enrolled. CDDP at a dose of 60 mg m^–2^was given first and followed by CPT-11 at a dose of 50 mg m^–2^. Both drugs were given by 1-hour infusion on days 1 and 8, and repeated every 4 weeks up to 4 cycles. 42 patients were evaluated for response and 44 for survival and toxicity. 20 patients (48%: 95% confidence interval 32–63%) achieved an objective response. The median duration of responses was 8 months, and the median survival time and the 1-year survival rate were 12.5 months and 56.8%, respectively. Major toxicities were neutropenia and diarrhoea. Grade 3 or 4 neutropenia occurred in 70.5% of the patients and one patient died of sepsis. Grade 3 or 4 diarrhoea was experienced in 25.0%, but manageable by conventional therapy. In conclusion, fractionated administration of CPT-11 and CDDP was highly effective for advanced NSCLC with manageable toxicities. © 2001 Cancer Research Campaign http://www.bjcancer.com


*British Journal of Cancer* (2001) **85**(1), 9–13
© 2001 Cancer Research Campaign
doi: 10.1054/ bjoc.2001.1861, available online at http://www.idealibrary.com on IDEAL®

### Fractionated administration of irinotecan and cisplatin for treatment of non-small-cell lung cancer: a phase II study of Okayama Lung Cancer Study Group

**H Ueoka[1], M Tanimoto[1], K Kiura[1], M Tabata[1], N Takigawa[1], Y Segawa[2], I Takata[2], K Eguchi[2], N Okimoto[3], S Harita[4], H Kamei[5], T Shibayama[6], Y Watanabe[7], S Hiraki[7] and M Harada[1]**

[1]Department of Internal Medicine II, Okayama University Medical School, 2-5-1 Shikatacho, Okayama 700-8558, Japan; [2]Department of Respiratory Medicine, National Shikoku Cancer Center; [3]Department of Internal Medicine, Kawasaki Medical School, Kawasaki Hospital; [4]Department of Internal Medicine, Chugoku Central Hospital; [5]Department of Internal Medicine, Sumitomobesshi Hospital; [6]Department of Internal Medicine, Okayama Health Foundation Hospital; [7]Department of Internal Medicine, Okayama Red Cross General Hospital

Summary A phase II study of fractionated administration of irinotecan (CPT-11) and cisplatin (CDDP) in patients with non-small-cell lung cancer (NSCLC) was conducted. Between January 1996 and January 1998, 44 previously untreated patients with stage IIIB or IV NSCLC were enrolled. CDDP at a dose of 60 mg $m^{-2}$ was given first and followed by CPT-11 at a dose of 50 mg $m^{-2}$. Both drugs were given by 1-hour infusion on days 1 and 8, and repeated every 4 weeks up to 4 cycles. 42 patients were evaluated for response and 44 for survival and toxicity. 20 patients (48%: 95% confidence interval 32–63%) achieved an objective response. The median duration of responses was 8 months, and the median survival time and the 1-year survival rate were 12.5 months and 56.8%, respectively. Major toxicities were neutropenia and diarrhoea. Grade 3 or 4 neutropenia occurred in 70.5% of the patients and one patient died of sepsis. Grade 3 or 4 diarrhoea was experienced in 25.0%, but manageable by conventional therapy. In conclusion, fractionated administration of CPT-11 and CDDP was highly effective for advanced NSCLC with manageable toxicities. © 2001 Cancer Research Campaign http://www.bjcancer.com

**Keywords**: phase II study; irinotecan; cisplatin; fractionated administration; non-small-cell lung cancer

Recent meta-analysis of randomized trials comparing cisplatin (CDDP)-based chemotherapy with best supportive care in patients with advanced non-small cell lung cancer (NSCLC) demonstrated some degree of survival benefit for CDDP-based chemotherapy (Grilli et al, 1993; Souquet et al, 1993; Non-small Cell Lung Cancer Collaborative Group, 1995). However, this benefit of improved survival was extremely limited. In one previous report, an objective response rate, a median survival time (MST), and a 1-year survival rate of advanced NSCLC patients receiving CDDP-based chemotherapy averaged approximately 25%, 6 months, and less than 20%, respectively (Johnson, 2000). In an attempt to improve these disappointing treatment outcomes of advanced NSCLC, several newly developed agents such as vinorelbine, taxans, gemcitabine and topoisomerase I inhibitors have been introduced in the 1990s (Bunn and Kelly, 1998).

Irinotecan (CPT-11) is a semisynthetic derivative of camptothecin that is metabolized in vivo to an active metabolite, SN-38, and exerts its cytotoxic activity by inhibiting a nuclear enzyme topoisomearase I (Kunimoto et al, 1987; Hsiang and Liu, 1988). CPT-11 has been shown to have a remarkable activity as a single agent for NSCLC (Negoro et al, 1991; Fukuoka et al, 1992). Since CDDP, a recent key drug in the treatment of NSCLC, has a different mechanism of action and a toxicity profile from CPT-11, and furthermore synergism between these 2 drugs was demonstrated in vitro (Kudoh et al, 1993), combination of CDDP and CPT-11 has been studied (Masuda et al, 1992, 1994, 1998; DeVore et al, 1999). In a Japanese multi-institutional study, CDDP was given on day 1 and CPT-11 on days 1, 8 and 15. An objective response rate in the study remained to be about 50%, because dose-escalation was prevented by diarrhoea and leukopenia which were mainly caused by CPT-11 (Masuda et al, 1992, 1994, 1998). In order to take maximum advantage of the synergistic effects between CDDP and CPT-11 and to reduce the dose of CPT-11, Okayama Lung Cancer Study Group planned the fractionated administration in which both CDDP and CPT-11 were given on days 1 and 8, and has already performed a phase I study (Ueoka et al, 1999). The present phase II study was planned to investigate the effectiveness of this fractionated administration of CDDP and CPT-11. The primary objective of this study was to determine the response rate, and secondary objectives were to evaluate survival and safety of this combination.

*Received 16 October 2000*
*Revised 19 March 2001*
*Accepted 27 March 2001*

*Correspondence to:* H Ueoka

### MATERIALS AND METHODS

### Patient selection

Eligibility requirements for entry into this study were as follows: (1) histologically or cytologically proven NSCLC; (2) stage IIIB or IV; (3) no prior chemotherapy, radiotherapy or surgery; (4) performance status (PS) of 0–2 on the Eastern Cooperative Oncology Group (ECOG) scale (Oken et al, 1982); (5) age 75 years or less; (6) presence of bidimensionally measurable disease;

(7) adequate functional reserves of the kidney (creatinine clearance > 60 ml $min^{-1}$), liver (ALT, AST < twice the upper limit of normal), and bone marrow (a leukocyte count > 4000 $\mu l^{-1}$ and platelet count > 100 000 $\mu l^{-1}$); (8) no concomitant malignancies; and (9) a written form of informed consent.

Stage IIIB patients whose tumours were encompassible in a radical radiotherapy volume were excluded. Patients with symptomatic superior vena caval obstruction, cerebral metastases, or previous malignancy were also ineligible. The present study was approved by the ethical committee of the Okayama Lung Cancer Study Group.

### Evaluation

Staging procedures included complete medical history and physical examination, urinalysis, a complete blood cell count (CBC), standard blood chemistry profile, 24-hour creatinine clearance (Ccr), electrocardiogram, a chest radiograph, fiberoptic bronchoscopy, computerized tomographic (CT) scans of the chest and abdomen, magnetic resonance imaging of the brain, and radionuclide bone scan. CBC was repeated 2 or 3 times a week, and urinalysis, blood chemistry, 24-hour Ccr, and chest radiograph were assessed at least once a week after initial evaluation. CT scans of the chest were repeated once a treatment cycle. After the completion of chemotherapy, each patient was restaged with all the tests used during the initial work-up. Eligibility and response of the enrolled patients were assessed by extramural reviewers. Responses and toxicities were evaluated according to the criteria of ECOG (Oken et al, 1982).

### Treatment plan

Both CDDP and CPT-11 were given by 1-hour intravenous infusion with an infusion pump on days 1 and 8. CDDP at a dose of 60 mg $m^{-2}$ with 100 ml physiological saline was given first and CPT-11 at a dose of 50 mg $m^{-2}$ dissolved in 300 ml of physiological saline was then administered. Granisetron (3 mg) or ondansetron (4 mg) was administered intravenously just before CDDP administration and hydration consisting of 3000 ml of physiological saline was given after administration of CDDP and CPT-11. When grade 3 or higher leukopenia or neutropenia occurred, administration of recombinant human granulocyte colony stimulating factor (rhG-CSF) was permitted under the guidelines of the Japanese Ministry of Health and Welfare. Loperamide was used for treatment of diarrhoea. Patients were instructed to have loperamide at the first onset of diarrhoea. Usually, loperamide at a dose of 1 mg was given twice a day, and increased up to 4 mg $day^{-1}$ according to the severity of diarrhoea.

The treatment was repeated every 4 weeks up to 4 cycles unless disease progression occurred. If grade 4 haematological toxicity or grade 3 diarrhoea was observed in the previous cycle, the dose of CPT-11 was reduced by 10 mg $m^{-2}$ in the next cycle. The dose of CDDP was reduced by 10 mg $m^{-2}$ for development of grade 4 haematological toxicity or by 30 mg $m^{-2}$ for development of grade 3 renal toxicity. If leukocyte counts were less than 2000 $\mu l^{-1}$ or platelet counts less than 100 000 $\mu l^{-1}$ on day 8, both CDDP and CPT-11 were not administered. Before the next course was started, leukocyte and platelet counts had to be at least 3500 $\mu l^{-1}$ or more and 100 000 $\mu l^{-1}$ or more, respectively.

### Statistical methods

The primary endpoint of this study was to estimate the objective response rate. Sample size of this study was determined on the assumption that the expected response rate would be 70%, with a 95% confidence interval of ± 15%. Time to progression and overall survival were calculated from the date of initiation of therapy using the Kaplan–Meier method. Difference in survival between 2 groups was assessed using a log-rank test. Dose-intensity (DI) was calculated as follows; DI = actually administered dose (mg $m^{-2}$ 4-$week^{-1}$)/projected dose (cisplatin 120 mg $m^{-2}$ 4-$week^{-1}$, irinotecan 100 mg $m^{-2}$ 4-$week^{-1}$) × 100. Statistical analyses were performed using SPSS Base System™ and Advanced Statistics™ Programs (SPSS Inc, Chicago, IL, USA).

### RESULTS

### Patient characteristics

Between January 1996 and January 1998, 44 patients were enrolled. The median age was 61 years (range 41–75 years). 31 patients were men and 13 women. PS was 0 in 20 patients, 1 in 19 and 2 in 5. 24 patients had adenocarcinoma, 15 squamous cell carcinoma, 4 large cell carcinoma, and 1 adenosquamous cell carcinoma. Clinical stage was IIIB in 14 patients and IV in 30.

### Response and survival

Responses to chemotherapy according to clinical stage and histology are summarized in Table 1. According to intent-to-treat analysis, 20 of the 44 patients enrolled in the study achieved a partial response and objective response rate was 45% (95% confidence interval: CI 30.5–60.5%). Since response was not evaluated in 2 patients because of early death by sepsis on day 22 and cerebral infarction on day 7, response rate among 42 assessable patients was 48% (95% CI 32–63%). The median time required to achieve objective response was 7 weeks (range 2–13 weeks). There were no significant differences in response rates according to clinical stage (50% for stage IIIB patients and 43% for stage IV) or histology (46% for adenocarcinoma, 53% for squamous cell carcinoma, and 25% for large cell carcinoma). The median time to progression for all patients was 8 months (range 1–49 months).

By a median follow-up time of 24 months (range 1–51 months), 38 (86%) patients have died and only 6 (14%) were still alive. The survival curve for 44 patients according to clinical stage is shown in Figure 1. The MST for all patients was 12.5 months and 1-, 2- and 3-year survival rates were 56.8%, 27.2%, and 17.1%, respectively. According to clinical stage, survival of patients in

**Table 1** Response

| | No. of patients | PR (%) | 95% confidence interval |
|---|---|---|---|
| Total | 44 | 20 (45) | 31–61 |
| Stage IIIB | 14 | 7 (50) | 23–76 |
| IV | 30 | 13 (43) | 25–61 |
| Histology | | | |
| Adenocarcinoma | 24 | 11 (46) | 26–66 |
| Squamous cell carcinoma | 15 | 8 (53) | 29–77 |
| Large cell carcinoma | 4 | 1 (25) | 3–47 |
| Adenosquamous | 1 | 0 | |

© 2001 Cancer Research Campaign

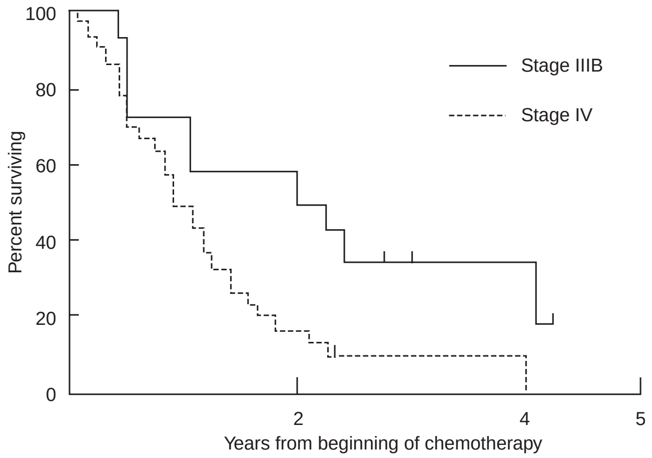

**Figure 1** Survival curves according to clinical stage

**Table 2** Toxicity

| | Grade | | | | | Grade 3/4 (%) |
|---|---|---|---|---|---|---|
| | 0 | 1 | 2 | 3 | 4 | |
| Anaemia | 2 | 7 | 16 | 12 | 7 | 43.2 |
| Leukopenia | 2 | 9 | 13 | 15 | 5 | 45.5 |
| Neutropenia | 2 | 1 | 10 | 9 | 22 | 70.5 |
| Thrombopenia | 6 | 15 | 5 | 12 | 6 | 40.9 |
| Nausea/vomiting | 2 | 10 | 21 | 10 | 1 | 25.0 |
| Appetite loss | 5 | 5 | 20 | 13 | 1 | 31.8 |
| Oral mucositis | 40 | 2 | 1 | 0 | 1 | 2.3 |
| Diarrhoea | 10 | 15 | 8 | 5 | 6 | 25.0 |
| Hair loss | 9 | 18 | 17 | | | |
| Neutropenic fever | 29 | 7 | 7 | | 1 | 2.3 |
| Renal damage | 33 | 6 | 4 | 1 | | 2.3 |
| Liver damage | 31 | 8 | 3 | 1 | 1 | 4.5 |

stage IIIB seemed to be superior to that in stage IV, but the difference was not significant (MST: 24.5 months vs 11.0 months, 1-, 2- and 3-year survival rates: 71.4% vs 50.0%, 34.3% vs 16.7%, 34.3% vs 10.0%, $P = 0.0672$).

### Toxicity

There was one treatment-related death. This patient died of sepsis due to severe neutropenia on day 22. Principal toxicities are listed in Table 2. The major haematological toxicity was neutropenia and grade 3 or 4 neutropenia was experienced in 70.5%, which was more frequent than expected from the results of a phase I study. However, no severe infectious complication except a fatal sepsis in one patient occurred. RhG-CSF was given following 39 (36%) of the 109 assessable courses. According to the number of the cycle, rhG-CSF was used in 18 of the 43 assessable courses at first cycle (42%, median duration of 9 days: range 3–18 days), 10 of the 33 at the second (30%, median duration of 5.5 days: range 3–21 days), 7 of the 21 at the third (33%, median duration of 9 days: range 5–24 days), and 4 of the 12 at the fourth (33%, median duration of 6.5 days: range 4–10 days). Though grade 3 or 4 thrombocytopenia was observed in 40.9%, no severe haemorrhagic complication was experienced. The major non-haematological toxicity was diarrhoea and grade 3 or 4 diarrhoea was encountered in 25.0%. Upper gastrointestinal toxicities were also frequent. But these toxicities were manageable and reversible by conventional therapy.

**Table 3** Dose intensity

| Course | No. of patients | Delivered dose/projected dose (%) | |
|---|---|---|---|
| | | Cisplatin | Irinotecan |
| #1 | 44 | 97.2 | 95.3 |
| #2 | 33 | 83.3 | 85.0 |
| #3 | 21 | 80.8 | 81.1 |
| #4 | 12 | 78.9 | 80.5 |

### Dose-intensity

12 patients (27%) completed the projected 4 cycles of chemotherapy. The second, third and fourth cycles of chemotherapy were not administered to 11 (25%), 23 (52%), and 32 (73%) patients, respectively. Reasons for not completing chemotherapy included no response ($n = 22$), patient's refusal ($n = 3$), toxicity or death ($n = 3$), disease recurrence ($n = 2$), and physician's discretion ($n = 2$). DI of each agent was shown in Table 3. DI was gradually decreased as cycles were repeated. However, in the fourth cycle, about 80% of the projected dose could be administered.

### DISCUSSION

We planned the present study to maximize the synergistic effect between CDDP and CPT-11. Therefore the schedule and sequence of administration of these 2 agents in the present study were different from those in the previous reports. Firstly, we conducted simultaneous administration of both drugs twice a cycle, while they were simultaneously given once a cycle in the previous reports. Secondly, though CPT-11 was given before CDDP administration in the Japanese multi-institutional study, we gave CDDP before CPT-11 administration, because this sequence was better than the inverted sequence in our in vitro study (Aoe et al, 1997).

In a phase I study, we determined the recommended dose of CPT-11 as 50 mg $m^{-2}$ when combined with CDDP at a dose of 60 mg $m^{-2}$ for the subsequent phase II study. At this dose level, only 2 of 7 patients (29%) developed grade 3 leukopenia and no patients developed severe non-haematological toxicities. Chemotherapy was repeated every 4 weeks, because it took 24 days (median days from initiation of chemotherapy) until recovery of neutrophil. The objective response rate, 76% for advanced NSCLC, was highly hopeful (Ueoka et al, 1999). Based on these results, we initiated the present phase II study, assuming the expected response rate to be 70%. The previously reported results of a combination of CDDP and CPT-11 for advanced NSCLC are summarized in Table4 (Masuda et al, 1998, 1999; DeVore et al, 1999; Niho et al, 1999). Though the response rate obtained in this phase II study was 45%, which was considerably lower than the expected response rate, it was comparable to the previously reported results. On the other hand, survival was fairly good. The MST (54 weeks) and 1-year survival rate (57%) were better than those reported previously with a combination of CPT-11 and CDDP (Masuda et al, 1998, 1999; DeVore et al, 1999; Niho et al, 1999).

The major haematological toxicity in the present study was neutropenia. Grade 3 and 4 neutropenia occurred in 71%, which was nearly comparable with those in the other reports, though severe infectious complications were rare. On the other hand,

© 2001 Cancer Research Campaign 

**Table 4** Phase II and III studies of a combination of cisplatin and irinotecan for advanced non-small cell lung cancer

| Investigator | No. of patients | CR + PR (%) | MST (week) | 1-year survival (%) | Grade 3/4 | |
|---|---|---|---|---|---|---|
| | | | | | Neutropenia | Diarrhoea |
| Masuda | 69 | 52 | 44 | 33 | 80 | 19 |
| DeVore | 52 | 29 | 43 | 37 | 46 | 17 |
| Niho | 98 | 29 | 46 | 43 | 62 | 13 |
| Masuda | 130 | 43 | 52 | 49 | 36 (gr 4) | 13 |
| Present study | 44 | 48 | 54 | 57 | 71 | 25 |

severe diarrhoea occurred in 25%, which was slightly higher than that in the previous reports (Masuda et al, 1998, 1999; DeVore et al, 1999; Niho et al, 1999). In the weekly administration of both CPT-11 and CDDP conducted by Kobayashi et al under similar consideration (Kobayashi et al, 1998), the most prominent toxicity was leukopenia, and diarrhoea was not a dose-limiting toxicity. CPT-11 has been considered as the major cause of diarrhoea in this combination. However, the incidence of severe diarrhoea was the most frequent in the present study, though DI of CPT-11 in the present study (25 mg $m^{-2}$ $week^{-1}$) was much lower than the multi-institutional study (45 mg $m^{-2}$ $week^{-1}$) and Kobayashi's study (45 mg $m^{-2}$). On the other hand, DI of CDDP in the present study (30 mg $m^{-2}$ $week^{-1}$) was higher than those in the multi-institutional study (20 mg $m^{-2}$ $week^{-1}$) and Kobayashi's study (25 mg $m^{-2}$). Thus, DI of CDDP may be related to the high incidence of diarrhoea in the present study. Furthermore, the sequence of administration of these 2 agents in the present study, CDDP before CPT-11, may develop higher AUC of CPT-11 and SN-38 than the inverted sequence. Therefore, high DI of CDDP and sequence of administration of these 2 drugs in the present study may have produced high AUC of CPT-11 and SN-38, and may have resulted in the increased incidence of severe diarrhoea. If the major obstacle of this fractionated administration of CPT-11 and CDDP is presumed to be diarrhoea during neutropenia, which may cause sepsis, effective prophylaxis of diarrhoea with loperamide will be important, and early administration of rhG-CSF may be necessary in the following study. Furthermore, prediction of patients so sensitive to CPT-11 and SN-38 as to develop severe diarrhoea also appears to be useful (Ando et al, 2000).

In conclusion, a fractionated administration of CPT-11 and CDDP was confirmed to be highly effective for advanced NSCLC with manageable toxicities. The treatment outcomes of the present study were superior or at least comparable to the results of previous multi-institutional trials of a combination of CPT-11 plus CDDP (Masuda et al, 1998, 1999; DeVore et al, 1999; Niho et al, 1999). Recently, superiority of 2-drug combinations of one of the newly developed agents, vinorelbine or paclitaxel, with CDDP to the representative CDDP-based chemotherapy in the 1980s such as CDDP plus vindesine or etoposide was reported in prospective randomized trials (Le Chevalier et al, 1994; Bonomi et al, 2000). However, no definitive conclusion was obtained in the 2 Japanese multi-institutional randomized trials comparing CPT-11 plus CDDP with CDDP plus vindesine (Masuda et al, 1999; Niho et al, 1999), although superiority of CPT-11 plus CDDP for stage IV patients was shown by the subset analysis of one trial (Masuda et al, 1999). Therefore further studies to establish the usefulness of the fractionated combination of CPT-11 and CDDP by comparing survival of this combination with that of a combination used in the Japanese multi-institutional trials will be warranted.

### REFERENCE

Ando Y, Saka H, Ando M, Sawa T, Muro K, Ueoka H, Yokoyama A, Saitoh S, Shimokata K and Hasegawa Y (2000) Polymorphisms of UDP-glucuronosyltransferase gene and irinotecan toxicity: a pharmacogenetic analysis. *Cancer Res* **60**: 6921–6926

Aoe K, Kiura K, Ueoka H, Tabata M, Chikamori K, Kohara H and Harada M (1997) Down-regulation of topoisomerase I induced by cisplatin. *Proc Am Assoc Cancer Res* **38**: 15

Bonomi P, Kim K, Fairclough d, Cella D, Kugler J, Rowinsky E, Jiroutek M and Johnson D (2000) Comparison of survival and quality of life in advanced non-small-cell lung cancer patients treated with two dose levels of paclitaxel combined with cisplatin versus etoposide with cisplatin: results of an Eastern Cooperative Oncology Group trial. *J Clin Oncol* **18**: 623–631

Bunn PA and Kelly K (1998) New chemotherapeutic agents prolong survival and improvequality of life in non-small cell lung cancer: A review of the literature and future directions. *Clin Cancer Res* **5**: 1087–1100

DeVore RF, Johnson DH, Crawford J, Garst J, Dimery IW, Eckardt J, Eckhardt SG, Elfring GL, Schaaf LJ, Hanover CK and Miller LL (1999) Phase II study of irinotecan plus cisplatin in patients with advanced non-small-cell lung cancer. *J Clin Oncol* **17**: 2710–2720

Fukuoka M, Niitani H, Suzuki A, Motomiya M, Hasegawa K, Nishiwaki Y, Kuriyama T, Ariyoshi Y, Negoro S, Masuda N, Nakajima S, Taguchi T for the CPT-11 Lung Cancer Study Group (1992) A phase II study of CPT-11, a new derivative of camptothecin, for previously untreated non-small-cell lung cancer. *J Clin Oncol* **10**: 16–20

Grilli R, Oxman AD and Julian JA (1993) Chemotherapy for advanced non-small-cell lung cancer: how much benefit is enough? *J Clin Oncol* **11**: 1866–1872

Hsiang YH and Liu LF (1988) Identification of mammalian DNA topoisomerase I as an intracellular target of the anticancer drug camptothecin. *Cancer Res* **48**: 1722–1726

Johnson DH (2000) Evolution of cisplatin-based chemotherapy in non-small cell lung cancer-A historical perspective and the Eastern Cooperative Oncology Group experience. *Chest* **117**: 133S–137S

Kobayashi K, Shinbara A, Kamimura M, Takeda Y, Kudo K, Kabe J, Hibino S, Hino M, Shibuya M and Kudoh S (1998) Irinotecan (CPT-11) in combination with weekly administration of cisplatin (CDDP) for non-small-cell lung cancer. *Cancer Chemother Pharmacol* **42**: 53–58

Kudoh S, Takada M, Masuda N, Nakagawa K, Itoh K, Kusunoki Y, Negoro S, Matsui K, Takifuji N, Morino H and Fukuoka M (1993) Enhanced antitumor efficacy of a combination of CPT-11, a new derivative of camptothecin, and cisplatin against human lung tumor xenografts. *Jpn J Cancer Res* **84**: 203–207

Kunimoto T, Nitta K, Tanaka T, Uehara N, Baba H, Takeuchi M, Yokokura T, Sawada S, Miyasaka T and Mutai M (1987) Antitumor activity of 7-ethyl-10-[4-(1-piperidino)-1-piperidino]carbonyloxy-camptothecin, a novel water-soluble derivative of camptothecin, against murine tumors. *Cancer Res* **47**: 5944–5947

Le Chevalier T, Brisgand D, Douillard JY, Pujol JL, Alberola V, Monnier A, Riviere A, Lianes P, Chomy P, Cigolari S, Gottfried M, Ruffie P, Panizo A, Gaspard M-H, Ravaioli A, Beseval M, Besson F, Martinez A, Berthaud P and Tursz T (1994) Randomized study of vinorelbine and cisplatin versus vindesine and cisplatin versus vinorelbine alone in advanced non-small-cell lung cancer: results of a European multicenter trial including 612 patients. *J Clin Oncol* **12**: 360–367

Masuda N, Fukuoka M, Takada M, Kusunoki Y, Negoro S, Matsui K, Kudoh S, Takifuji N, Nakagawa K and Kishimoto S (1992) CPT-11 in combination with cisplatin for advanced non-small-cell lung cancer. *J Clin Oncol* **10**: 1775–1780

Masuda N, Fukuoka M, Kudoh S, Kusunoki Y, Matsui K, Nakagawa K, Hirashima T, Tamanoi M, Nitta T, Yana T, Negoro S, Takifuji N and Takada M (1994) Phase I study of irinotecan and cisplatin with granulocyte colony-stimulating factor support for advanced non-small-cell lung cancer. *J Clin Oncol* **12**: 90–96

© 2001 Cancer Research Campaign

Masuda N, Fukuoka M, Fujita A, Kurita Y, Tsuchiya S, Nagao K, Negoro S, Nishikawa H, Katakami N, Nakagawa K and Niitani H (1998) A phase II trial of combination of CPT-11 and cisplatin for advanced non-small-cell lung cancer. CPT-11 Lung Cancer Study Group. *Br J Cancer* **78**: 251–256

Masuda N, Fukuoka M, Negoro S, Takada M, Sugiura T, Ohashi Y, Ariyoshi Y, H Niitani and the CPT-11 Lung Cancer Study Group (1999) Randomized trial comparing cisplatin (CDDP) and irinotecan (CPT-11) versus CDDP and vindesine (VDS) versus CPT-11 alone in advanced non-small cell lung cancer (NSCLC), a multicenter phase III study. *Proc Am Soc Clin Oncol* **18**: 459a

Negoro S, Fukuoka M, Masuda N, Takada M, Kusunoki Y, Matsui K, Takifuji N, Kudoh S, Niitani H and Taguchi T (1991) Phase I study of weekly intra venous infusion of CPT-11, a new derivative of camptothecin, in the treatment of advanced non-small-cell lung cancer. *J Natl Cancer Inst* **83**: 1164–1168

Niho S, Nagao K, Nishiwaki Y, Yokoyama A, Saijo N, Ohashi Y, Niitani H and the CPT-11 Lung Cancer Study Group (1999) Randomized multicenter phase III trial of irinotecan (CPT-11) and cisplatin (CDDP) versus CDDP and vindesine (VDS) in patients with advanced non-small cell lung cancer (NSCLC). *Proc Am Soc Clin Oncol* **18**: 492a

Non-small Cell Lung Cancer Collaborative Group (1995) Chemotherapy in non-small cell lung cancer: a meta-analysis using updated data on individual patients from 52 randomized clinical trials. *BMJ* **311**: 899–909

Oken MM, Creech RH, Tormey DC, Horton J, Davis TE, McFadden ET and Carbone PP (1982) Toxicity and response criteria of the Eastern Cooperative Oncology Group. *Am J Clin Oncol* **5**: 649–655

Souquet PJ, Chauvin F, Boissel JP, Cellerino R, Cormier Y, Ganz PA, Kaasa S, Pater JL, Quoix E and Rapp E (1993) Polychemotherapy in advanced non small cell lung cancer: a meta-analysis. *Lancet* **342**: 19–21

Ueoka H, Tabata M, Kiura K, Shibayama T, Gemba K, Segawa Y, Chikamori K, Yonei T, Hiraki S and Harada M (1999) Fractionated administration of irinotecan and cisplatin for treatment of lung cancer: a phase I study. *Br J Cancer* **79**: 984–990

*© 2001 Cancer Research Campaign*
